# Einfluss der MRT-Fusionsbiopsie auf Therapieempfehlungen: Wie verlässlich ist das Biopsieergebnis?

**DOI:** 10.1007/s00120-025-02738-8

**Published:** 2025-12-09

**Authors:** P. Schildhauer, M. Müller, A. S. Merseburger, S. M. Weiler, A. Fürschke, Y. Elser, A. Moderegger, H. E. Fender

**Affiliations:** 1 Klinik für Urologie, Universitätsklinikum Schleswig-Holstein, Campus Lübeck, Lübeck, Deutschland; 2 Experimental Psychology Unit, Helmut-Schmidt-Universität/Universität der Bundeswehr Hamburg, Hamburg, Deutschland; 3 Klinik für Radiologie, Universitätsklinikum Schleswig-Holstein, Campus Lübeck, Lübeck, Deutschland

**Keywords:** Prostatakarzinom, Stanzbiopsie, Prostate Imaging Reporting and Data System, Gleason-Score, Prostatektomie, Prostate cancer, Punch biopsy, Prostate Imaging Reporting and Data System, Gleason score, Prostatectomy

## Abstract

**Hintergrund:**

Die Detektion klinisch signifikanter Prostatakarzinome bleibt eine diagnostische Herausforderung. Die Kombination aus MRT-Fusionsbiopsie und systematischer Biopsie gilt als Standard in der Primärdiagnostik.

**Ziel der Arbeit:**

Untersucht wurden Unterschiede in den leitliniengerechten Therapieempfehlungen basierend auf der Histologie der MRT-Fusions- bzw. systematischen Biopsie sowie deren Übereinstimmung mit der Resektathistologie.

**Material und Methoden:**

Eingeschlossen wurden 476 Patienten mit Prostatabiopsie zwischen Januar 2022 und Dezember 2024. Primäre Endpunkte waren die histologischen Ergebnisse je Biopsiemethode und die daraus abgeleiteten Therapieempfehlungen. In einer Subgruppe von 57 Patienten (115 Läsionen) mit radikaler Prostatektomie wurde die Übereinstimmung zwischen Biopsie- und Resektathistologie in Abhängigkeit vom PI-RADS-Score analysiert. Die Auswertung erfolgte mittels Binomialtest, McNemar-Test, Cohens Kappa und logistischer Regression (*p* < 0,05).

**Ergebnisse:**

Das kombinierte Verfahren detektierte signifikant mehr klinisch relevante Karzinome als die systematische Biopsie (41,7 % vs. 27,9 %; *p* < 0,001) und führte häufiger zu kurativen Therapieempfehlungen (36,0 % vs. 26,2 %; *p* < 0,001). Auch gegenüber der MRT-Fusionsbiopsie allein war die Rate kurativer Empfehlungen höher (36,0 % vs. 32,8 %; *p* < 0,001). Die Übereinstimmung des Gleason-Scores mit dem Resektat war bei PI-RADS-5-Läsionen am höchsten (κ = 0,294) und signifikant höher als bei PI-RADS‑3 (*p* = 0,029).

**Schlussfolgerung:**

Das kombinierte Biopsieverfahren erhöht die Rate leitliniengerechter kurativer Therapieempfehlungen. Der zusätzliche Nutzen der systematischen Biopsie bleibt jedoch gering. Ein höherer Übereinstimmungsgrad zwischen Biopsie- und Resektathistologie zeigte sich nur bei PI-RADS-5-Läsionen.

**Graphic abstract:**

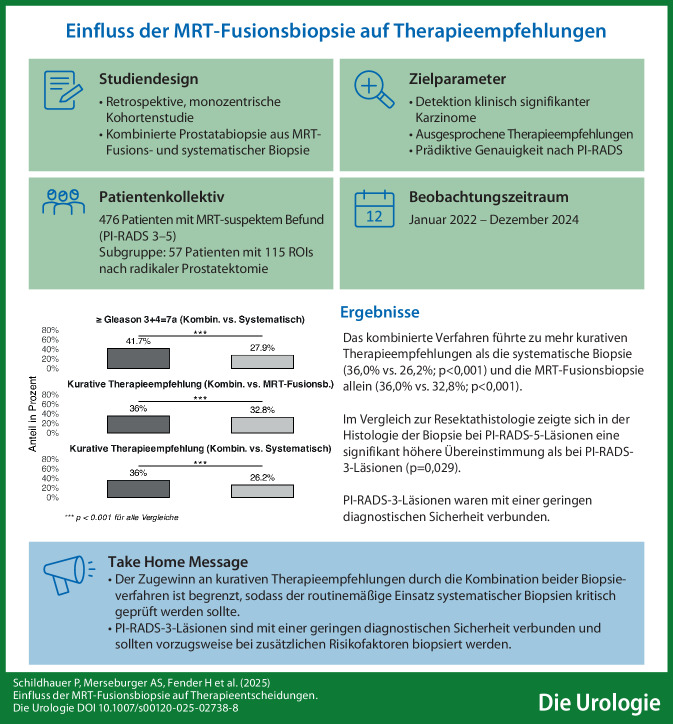

## Abstract (en)

**Background:**

The detection of clinically significant prostate cancer remains a diagnostic challenge. The combination of magnetic resonance imaging (MRI) fusion biopsy and systematic biopsy is currently considered standard in primary diagnostics.

**Objectives:**

The study examined differences in guideline-based treatment recommendations derived from the histology of MRI fusion versus systematic biopsy and their concordance with prostatectomy histology.

**Materials and methods:**

A total of 476 patients who underwent prostate biopsy between January 2022 and December 2024 were included. Primary endpoints were histological classification by biopsy method and the resulting treatment recommendations. In a subgroup of 57 patients (115 lesions) who underwent radical prostatectomy, concordance between biopsy and surgical histology was analyzed based on Prostate Imaging Reporting and Data System (PI-RADS) scores. Statistical analyses included binomial test, McNemar’s test, Cohen’s κ, and binary logistic regression (significance level *p* < 0.05).

**Results:**

The combined approach detected significantly more clinically relevant cancers than systematic biopsy alone (41.7% vs. 27.9%; *p* < 0.001) and led to more curative treatment recommendations (36.0% vs. 26.2%; *p* < 0.001). Compared with MRI fusion biopsy alone, it also yielded a higher rate of curative recommendations (36.0% vs. 32.8%; *p* < 0.001). Concordance of Gleason scores with prostatectomy findings was highest for PI-RADS 5 lesions (κ = 0.294) and significantly higher than for PI-RADS 3 (*p* = 0.029).

**Conclusion:**

The combined biopsy approach increases the rate of guideline-concordant curative treatment recommendations. However, the added diagnostic value of systematic biopsy remains limited. Higher concordance between biopsy and prostatectomy histology was observed only in PI-RADS 5 lesions.

## Hinführung zum Thema

Die MRT(Magnetresonanztomographie)-Fusionsbiopsie erzielt höhere Detektionsraten klinisch signifikanter Tumoren als die systematische Biopsie, deren zusätzlicher Nutzen umstritten ist. Der PI-RADS(Prostate Imaging-Reporting and Data System)-Score dient dabei als MRT-basiertes Bewertungssystem zur Einschätzung der Karzinomwahrscheinlichkeit. Diese Studie vergleicht die diagnostische Genauigkeit in Abhängigkeit vom PI-RADS-Score sowie die aus beiden Biopsieverfahren abgeleiteten Therapieempfehlungen.

## Hintergrund und Fragestellung

Das Prostatakarzinom (PCa) zählt zu den häufigsten soliden Tumoren des Mannes und ist trotz hoher Überlebensraten weltweit eine bedeutende Ursache tumorassoziierter Mortalität [8, 13, 17, 18]. Metaanalysen und randomisierte kontrollierte Studien belegen die Überlegenheit der MRT-Fusionsbiopsie gegenüber der systematischen Biopsie bei der Detektion klinisch signifikanter Karzinome (csPCa; [5, 6, 21]). Dennoch weisen verschiedene Arbeiten darauf hin, dass die ergänzende Durchführung der systematischen Biopsie weiterhin von Nutzen sein kann [11, 16, 20].

Entscheidend ist jedoch nicht nur die Detektion von csPCa, sondern auch der Einfluss der Biopsiebefunde auf die Therapieempfehlungen. Daher untersucht diese Studie, ob sich die aus der Histologie der MRT-Fusions- und der systematischen Biopsie abgeleiteten Therapieempfehlungen unterscheiden.

Die bislang unzureichend untersuchte Übereinstimmung der Biopsiebefunde mit der endgültigen Tumorhistologie nach radikaler Prostatektomie (RPE) erschwert außerdem eine fundierte Bewertung der diagnostischen Verfahren. Diamand et al. und Andras et al. zeigten beispielsweise, dass das kombinierte Biopsieverfahren aus MRT-Fusionsbiopsie und systematischer Biopsie die Vorhersage des endgültigen Tumorgrades nach RPE verbessern kann [3, 4]. Diese Studie soll die bestehende Evidenz zur Prostatabiopsie erweitern, indem die diagnostische Genauigkeit der MRT-Fusionsbiopsie in Abhängigkeit vom PI-RADS-Score sowie der systematischen Biopsie mit der endgültigen Tumorhistologie nach RPE verglichen wird.

## Studiendesign und Untersuchungsmethoden

Im Rahmen dieser retrospektiven, monozentrischen Studie wurden 476 Patienten eingeschlossen, die zwischen Januar 2022 und Dezember 2024 ein kombiniertes Biopsieverfahren (systematische Biopsie plus MRT-Fusionsbiopsie) der Prostata erhielten. Die Datenerhebung erfolgte anonymisiert unter Beachtung der geltenden Datenschutzrichtlinien. Die Studie wurde durch das zuständige Ethikkomitee genehmigt (Aktenzeichen: 2025-091). Teile der Methodik basieren auf einer früheren Analyse unserer Arbeitsgruppe (Manuskript unter Überprüfung).

Die präbioptische Auswertung der mpMRT-Aufnahmen erfolgte in der radiologischen Abteilung gemäß einem standardisierten Protokoll. Als „region of interest“ (ROI) wurden ausschließlich Läsionen mit einem PI-RADS-Score von 3–5 definiert; für jede ROI wurden Anzahl der Stanzen und die PI-RADS-Klassifikation (Version 2.1) dokumentiert. Insgesamt wurden die Biopsien von 20 Operateuren durchgeführt. Die Markierung der Zielregionen im MRT erfolgte durch die radiologische Abteilung gemäß den jeweiligen Fusionsprotokollen unserer Einrichtung. Alle Untersuchungen wurden in unserer Universitätsklinik durch einen Q2-zertifizierten Radiologen validiert und bei unzureichender Bildqualität mittels Kurzprotokoll-MRT ergänzt.

Die transperineale Biopsie wurde unter Echtzeitnavigationskontrolle mit dem MRT-Ultraschallfusionssystem BioJet (Rev 3.0, Medical Targeting Technologies GmbH) durchgeführt (Ultraschallgerät: BK Medical, Typ 2202). Im Mittel wurden pro ROI drei gezielte MRT-Fusionsbiopsien entnommen, ergänzt durch eine systematische Biopsie gemäß internem Standard (12-Kern-Raster, bei dem jeweils 6 Stanzen aus der rechten und linken Prostatahälfte entnommen wurden; jeweils lateral und medial aus Apex, mittlerem Abschnitt und Basis). Die histopathologische Analyse umfasste die Bestimmung des Gleason-Scores, die Quantifizierung des maximalen Tumoranteils (%), sowie die Erfassung kribriformer und intraduktaler Karzinomanteile. Klinisch signifikante Tumoren wurden gemäß international etablierter Definition als Karzinome mit einem Gleason-Score ≥ 3 + 4 = 7a klassifiziert [2, 7, 12].

Auf der Grundlage des kombinierten Biopsieverfahrens formulierte die interdisziplinäre Tumorkonferenz Therapieempfehlungen, diese wurden dokumentiert. Zur Auswertung der Therapieentscheidungen wurden zusätzlich hypothetische Empfehlungen basierend auf den getrennten Ergebnissen der MRT-Fusionsbiopsie und der systematischen Biopsie abgeleitet. Diese hypothetischen leitliniengerechten Empfehlungen basierten auf der aktuellen S3-Leitlinie Prostatakarzinom und wurden von einem erfahrenen Facharzt und Leiter des urologischen Tumorboards erstellt [21].

Zur systematischen Bewertung wurden die Befunde in fünf therapiebezogene Gruppen eingeteilt (s. Tab. 1).

**Tab. 1:** Zuordnung von Therapieempfehlungen zu histologischen Befunden

Therapieempfehlung	Histologischer Befund
1. Kein Anhalt für Malignität	Kein Anhalt für Malignität
2. Erneute Biopsieempfehlung bei Nachweis von PIN oder ASAP	ASAP, PIN
3. Aktive Überwachung	3 + 3 = 6, 3 + 4 = 7a^a^
4. Kurative interventionelle Therapie bei lokalisiertem Karzinom	3 + 4 = 7a*, 4 + 3 = 7b
5. High-risk-Befund: Staging und potenziell kurative Therapie	3 + 5 = 8, 5 + 3 = 8, 4 + 4 = 8, 4 + 5 = 9, 5 + 4 = 9, 5 + 5 = 10

*ASAP* „atypical small acinar proliferation“, *PIN* „prostatic intraepithelial neoplasia“

^a^Die Zuordnung von 3 + 4 = 7a zur aktiven Überwachung erfolgte gemäß S3-Leitlinie, Version 7, bei günstiger Risikokonstellation (z. B. kein kribriformes und/oder intraduktales Wachstum und einen Anteil Gleason-Muster 4 unter 10 %; [21])

### Subanalyse der Patienten mit Prostatektomie

Es wurden 115 ROI von 57 Patienten eingeschlossen, die zwischen Januar 2022 und Oktober 2024 nach kombinierter Biopsie radikal prostatektomiert wurden. Die Biopsatbefunde wurden mit der finalen RPE-Histologie verglichen. Die Ergebnisse wurde in 3 klinisch relevante Subgruppen unterteilt: (1) niedriggradige Karzinome (3 + 3 = 6) mit Empfehlung zur aktiven Überwachung, (2) intermediärgradige Karzinome (≥ 3 + 4 = 7a) mit kurativer Therapieindikation ohne zusätzliches Staging sowie (3) hochgradige Karzinome (≥ 4 + 4 = 8) mit Empfehlung zum Staging und ggf. kurativer Therapie.

### Statistik

Die statistische Auswertung erfolgte mit Jamovi [19], basierend auf R [15]. Zur Analyse dichotomer Variablen wurde der Binomialtest eingesetzt. Unterschiede in abhängigen dichotomen Stichproben wurden mit dem McNemar-Test geprüft. Der Einfluss ordinaler oder kategorialer Prädiktoren auf die Detektion csPCa (≥ Gleason 3 + 4 = 7a) wurde mittels binärer logistischer Regression analysiert. Die Vergleichbarkeit der diagnostischen Genauigkeit systematischer und MRT-gestützter Biopsien in Bezug auf die Übereinstimmung mit der Prostatektomiehistologie wurde anhand von Cohen’s Kappa sowie durch die Berechnung der Sensitivitäten in definierten Subgruppen bewertet. Das Signifikanzniveau wurde auf α = 0,05 festgelegt; *p*-Werte < 0,05 galten als statistisch signifikant.

## Ergebnisse

### Patientencharakteristika und klinische Basisdaten

Die Tab. 2 zeigt die demographischen und klinischen Charakteristika der Studienpopulation, einschließlich Alter, PSA-Wert und Prostatavolumen anhand der MRT-Befunde.

**Tab. 2:** Tabelle der demographischen und klinischen Daten

	Alter	PSA-Wert (ng/ml)	Prostatavolumen MRT (ml)
*N*	476	468	429
Mittelwert	66,8	8,5	64,6
Median	67	6,8	56
Standardabweichung	7,5	6,6	34
Minimum	47	0,2	12
Maximum	87	55,5	240

Das kombinierte Biopsieverfahren weist eine höhere Detektionsrate csPCa auf.

Das kombinierte Biopsieverfahren (systematisch + MRT-Fusionsbiopsie) identifizierte bei 198 Patienten (41,7 %) ein csPCa (≥ Gleason 3 + 4 = 7a). Im Vergleich zur alleinigen systematischen Biopsie, die bei 133 Patienten (27,9 %) ein csPCa detektierte, war dies ein signifikanter Unterschied (McNemar-Test: χ^2^ = 63,1; *p* < 0,001). Das 95 %-Konfidenzintervall (KI) für die Detektionsrate des kombinierten Biopsieverfahrens lag bei 37,2–46,3 %, verglichen mit 24,0–32,2 % für die systematische Biopsie.

Das *kombinierte Biopsieverfahren* führte in 10 % bzw. 3 % mehr Fällen zu kurativen Therapieempfehlungen*.*

**Abb. 1:**
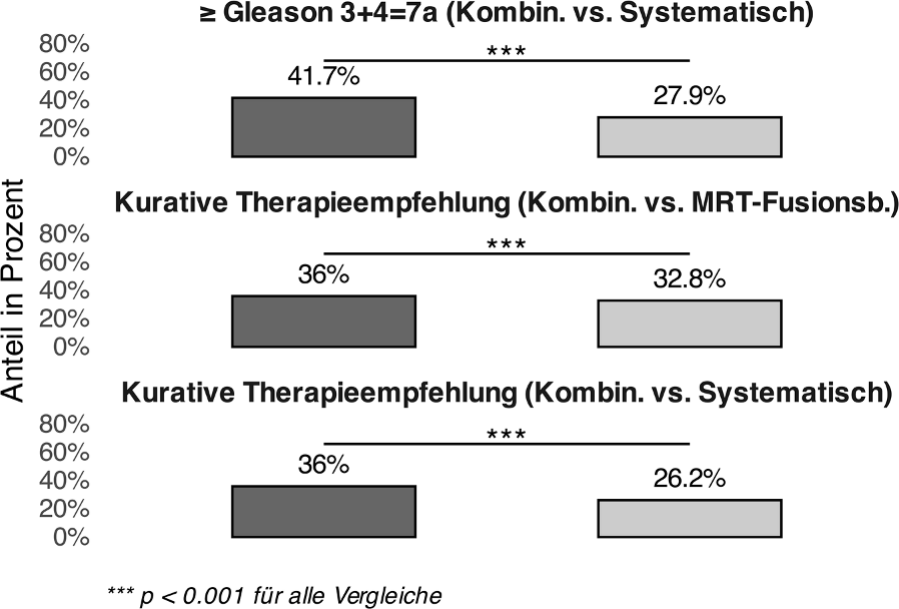
Vergleich kurativer Therapieempfehlungen und Karzinomdetektion nach Biopsiemethode. *Anmerkung:* Vergleich der Anteile (in %) an klinisch signifikanten Prostatakarzinomen (csPCa; ≥ Gleason 3 + 4 = 7a) und kurativen Therapieempfehlungen zwischen kombiniertem Biopsieverfahren (*kombininiert* = MRT-Fusionsbiopsie + systematische Biopsie, *schwarz*), MRT-Fusionsbiopsie (MRT-Fusionsbiopsie) und systematischer Biopsie (jeweils *grau*)

Insgesamt erhielten 36 % der Patienten (*n* = 169) gemäß der Empfehlung des interdisziplinären Tumorboards auf Grundlage der Ergebnisse des kombinierten Biopsieverfahrens eine kurative Therapieempfehlung. Wären ausschließlich die Ergebnisse der systematischen Biopsie berücksichtigt worden, hätte lediglich in 26,2 % der Fälle (*n* = 124) eine kurative Therapieempfehlung ausgesprochen werden sollen (dies entspricht den Zuordnungen der Patienten zu Gruppe 4 und 5). Bei ausschließlicher Berücksichtigung der MRT-Fusionsbiopsie hätte in 32,8 % der Fälle (*N* = 156) eine kurative Therapieempfehlung resultieren sollen. Das kombinierte Biopsieverfahren führte signifikant häufiger zu einer kurativen Therapieempfehlung als die alleinige systematische Biopsie (36 % vs. 26,2 %; McNemar-Test: χ^2^ = 45,0; *p* < 0,001). Das 95 %-KI für die kurative Empfehlung lag bei 31,6–40,5 % für das kombinierte Biopsieverfahren und bei 22,3–30,4 % für die systematische Methode. Auch im Vergleich zur alleinigen MRT-Fusionsbiopsie resultierte das kombinierte Biopsieverfahren signifikant häufiger in einer kurativen Therapieempfehlung (36 % vs. 32,8 %; McNemar-Test: χ^2^ = 14,2; *p* < 0,001) (s. Abb. 1). Das 95 %-KI lag bei 31,6–40,5 % für das kombinierte Biopsieverfahren und bei 28,6–37,3 % für die alleinige MRT-Fusionsbiopsie. Bei fünf Fällen (*n* = 5) war die Therapieempfehlung des interdisziplinären Tumorboards retrospektiv nicht rekonstruierbar, sodass diese Datensätze von der entsprechenden Auswertung ausgeschlossen wurden.

### Jährliche Fallzahlen und Therapieempfehlungen

Die Tab. 3 zeigt die jährliche Verteilung der Fallzahlen sowie der durch das Tumorboard empfohlenen kurativen Therapieempfehlungen. In 5 Fällen (*n* = 5) konnte die Therapieempfehlung retrospektiv nicht rekonstruiert werden, weshalb diese Datensätze von der Auswertung ausgeschlossen wurden.

**Tab. 3:** Jahresbezogene Auswertung der kurativen Therapieempfehlung des interdisziplinären Tumorboards

Jahr	Anzahl (*n*)	Kurative Therapieempfehlungen (*n*)	Kurative Therapieempfehlungen (%)
2022	127	53	41,7
2023	173	62	35,8
2024	176	54	30,7

*Anmerkung. *In 5 Fällen (*n* = 5) konnte die Therapieempfehlung retrospektiv nicht rekonstruiert werden

Jeder dritte Patient erhielt durch die MRT-Fusionsbiopsie ein *„Upgrading“ einer Therapieempfehlung.*

Die MRT-Fusionsbiopsie ermöglichte in 33,4 % der Fälle (*n* = 159) eine Änderung der Therapieempfehlung im Vergleich zur alleinigen systematischen Biopsie. Grundlage war zunächst die Einteilung in Therapiegruppen anhand der histopathologischen Befunde der systematischen Biopsie (s. Tab. 1). Nach Hinzunahme der Histologie der MRT-Fusionsbiopsie wurde erneut eine Therapiezuordnung vorgenommen und etwaige Änderungen dokumentiert. Berücksichtigt wurden ausschließlich relevante Therapieanpassungen im Sinne eines Upstagings bzw. einer neu empfohlenen kurativen Therapie.

### Subgruppenanalyse der Patienten mit radikaler Prostatektomie

#### Validität der Biopsieverfahren im Vergleich zur Resektathistologie

**Abb. 2:**
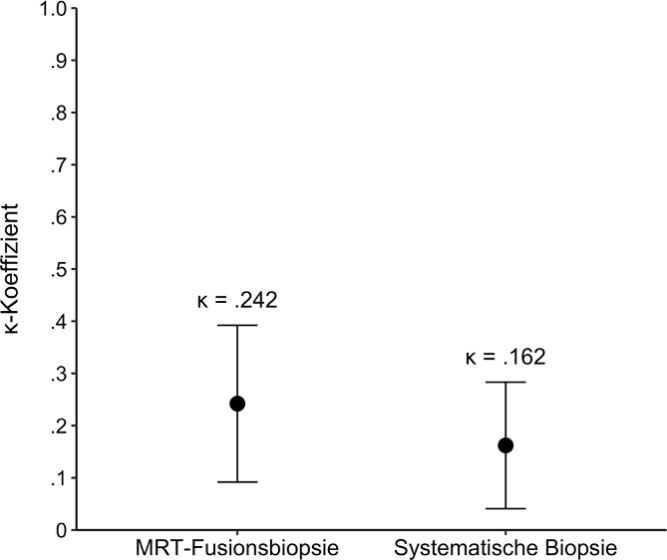
Übereinstimmung (Cohen’s Kappa) der MRT-Fusions- und systematischen Biopsie mit der Tumorhistologie nach radikaler Prostatektomie

Die Gesamtgenauigkeit der MRT-Fusionsbiopsie betrug 69,2 % (95 %-KI: 54,9–81,3 %), die der systematischen Biopsie 59,2 % (95 %-KI: 44,2–73,0 %). Die Bewertung erfolgte anhand der korrekten Zuordnung zu drei Subgruppen: niedriggradige Karzinome (Gleason 3 + 3 = 6; *n* = 3), intermediärgradige (≥ 3 + 4 = 7a; *n* = 49) und hochgradige Karzinome (≥ 4 + 4 = 8; *n* = 5). Der Cohen’s Kappa-Koeffizient (κ) zeigte für die MRT-Fusionsbiopsie eine leichte bis moderate Übereinstimmung (κ = 0,242), für die systematische Biopsie eine sehr geringe Übereinstimmung (κ = 0,162; s. Abb. 2). Die Differenz der Kappa-Werte (∆κ = 0,079; 95 %-KI: −0,113 bis 0,272) war statistisch nicht signifikant.

#### Sensitivität und prädiktiver Wert in Abhängigkeit vom Tumorgrad

**Abb. 3:**
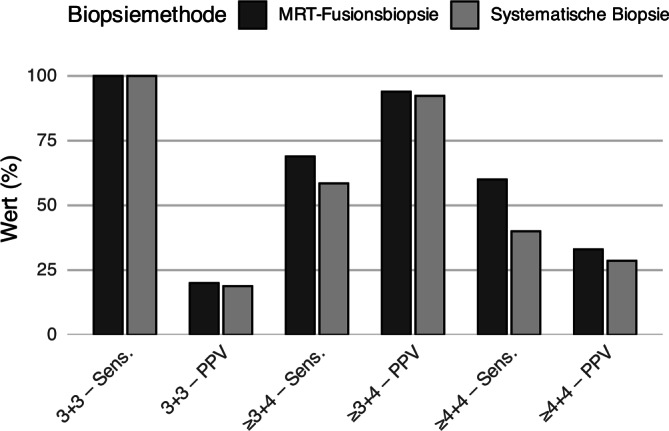
Vergleich der Sensitivität und des positiven prädiktiven Werts (PPV) der MRT-Fusionsbiopsie und der systematischen Biopsie. *Anmerkung: Schwarze Balken* stellen die Ergebnisse der MRT-Fusionsbiopsie dar, *graue Balken* die der systematischen Biopsie. Dargestellt sind Sensitivität und positiver prädiktiver Wert in den drei Tumorklassifikationen: niedriggradig (Gleason 3 + 3 = 6), intermediärgradig (≥ 3 + 4 = 7a) und hochgradig (≥ 4 + 4 = 8)

Alle in die Subgruppenanalyse eingeschlossenen Patienten zeigten ein histologisch gesichertes PCa im Resektionspräparat nach radikaler Prostatektomie. In der Subgruppenübersicht wiesen beide Biopsieverfahren unterschiedliche Sensitivitäten und positive prädiktive Werte auf (s. Abb. 3). Bei 5 Patienten mit radikaler Prostatektomie ergab die Resektathistologie eine Tumorhistologie, die ein präoperatives Staging erforderlich gemacht hätte. Die systematische Biopsie identifizierte diese Konstellation in 2 Fällen (40 %), die MRT-Fusionsbiopsie in 3 Fällen (60 %).

#### Übereinstimmung von Biopsie und Resektat nach PI-RADS

**Abb. 4:**
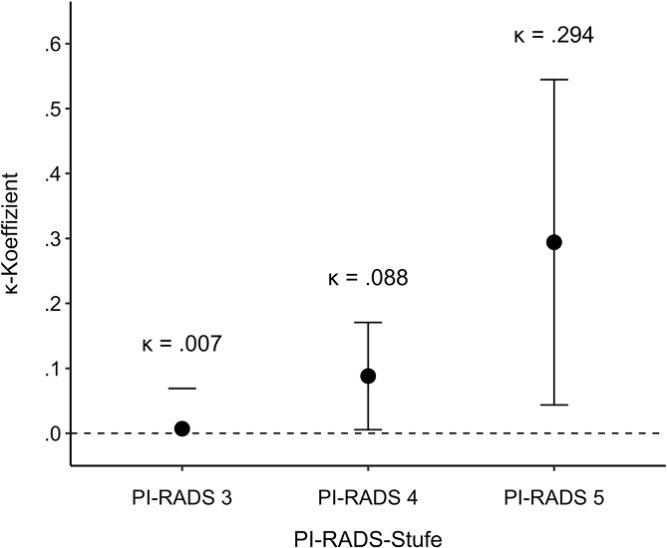
Verlauf der Cohen’s Kappa-Koeffizienten in Abhängigkeit vom PI-RADS-Score

Für PI-RADS-3-Läsionen (*n* = 35) lag der Cohen’s Kappa bei κ = 0,007 (z = 0,223, *p* = 0,824), was auf keine signifikante Übereinstimmung zwischen Biopsie- und Resektatbefund hinweist. Bei PI-RADS-4-Läsionen (*n* = 61) betrug der Kappa-Wert κ = 0,088 (z = 2,09, *p* = 0,037) und zeigte eine geringe, statistisch signifikante Übereinstimmung. Für PI-RADS-5-Läsionen (*n* = 19) wurde mit κ = 0,294 (z = 2,30, *p* = 0,021) die höchste Übereinstimmung innerhalb der Subgruppen beobachtet (s. Abb. 4).

#### Signifikant höhere Biopsie-Resektat-Übereinstimmung bei PI-RADS 5

Der Vergleich der Übereinstimmung zwischen Biopsie- und Resektatbefund (s. Tab. 4) ergab eine signifikant höhere Übereinstimmung bei PI-RADS-5-Läsionen im Vergleich zu PI-RADS-3-Läsionen (*p* = 0,029).

**Tab. 4:** Vergleich der Übereinstimmung zwischen Biopsie- und Resektatbefund nach PI-RADS-Kategorien (Prostate Imaging Reporting and Data System)

Vergleich	Differenz	z‑Wert	*p*-Wert
PI-RADS 4–PI-RADS 3	0,081	1,54	0,124
PI-RADS 5–PI-RADS 3	0,287	2,18	0,029
PI-RADS 5–PI-RADS 4	0,206	1,53	0,126

## Diskussion

Das kombinierte Biopsieverfahren detektierte signifikant häufiger klinisch relevante PCa (Gleason-Score ≥ 3 + 4 = 7a) als die alleinige systematische Biopsie (*p* < 0,001). Die Ergebnisse bestätigen frühere Studien, die die Überlegenheit des kombinierten Biopsieverfahrens bei der Detektion csPCa beschreiben [1, 5, 9, 10]. Da histopathologische Befunde nicht zwingend eine kurative Therapieempfehlung nach sich ziehen, etwa bei Karzinomen mit Gleason-Score 3 + 4 = 7a, die unter bestimmten Voraussetzungen ebenfalls einer Active Surveillance zugeführt werden können, ist der Einfluss der Biopsiemethode auf die Rate kurativer Therapieempfehlungen von klinischer Relevanz. Das kombinierte Biopsieverfahren führte signifikant häufiger zu kurativen Therapieempfehlungen als die alleinige systematische oder MRT-Fusionsbiopsie (*p* < 0,001). Durch die ergänzende Durchführung der systematischen Biopsie zur MRT-Fusionsbiopsie stieg die Rate kurativer Empfehlungen jedoch lediglich um 3 %. Daraus ergibt sich eine „number needed to test“ von 33, was bedeutet, dass bei 33 zusätzlich zur MRT-Fusionsbiopsie systematisch biopsierten Patienten in einem weiteren Fall eine kurative Therapieempfehlung ausgesprochen wurde. Angesichts dieses geringen absoluten Zugewinns erscheint der routinemäßige additive Einsatz der systematischen Biopsie im Rahmen der Primärdiagnostik kritisch zu hinterfragen.

Die MRT-Fusionsbiopsie wies eine höhere Übereinstimmung mit dem postoperativen Histologiebefund nach RPE auf als die systematische Biopsie, allerdings ohne statistisch signifikanten Unterschied. Diamand et al. berichten über signifikante Unterschiede in der Übereinstimmung zwischen MRT-Fusionsbiopsie und systematischer Biopsie [4]. Ein direkter Vergleich mit unseren Ergebnissen ist aufgrund unterschiedlicher Studiendesigns nur eingeschränkt möglich.

Die systematische Biopsie zeigte insgesamt eine geringere Sensitivität und einen niedrigeren positiven prädiktiven Wert als die MRT-Fusionsbiopsie. Bei hochgradigen Tumoren (Gleason ≥ 4 + 4 = 8) war die diagnostische Qualität beider Verfahren eingeschränkt, was das Risiko von Fehldiagnosen erhöht.

Die Übereinstimmung zwischen Biopsie- und Resektatbefund nimmt mit steigender PI-RADS-Kategorie zu, was mit den Befunden von Andras et al. konsistent ist [3]. Bei PI-RADS-3-Läsionen ist die Korrelation nahezu nicht vorhanden, was die diagnostische Unsicherheit dieser Befundkategorie widerspiegelt. Diese Kategorie wird in der neuen Konsultationsfassung der S3-Leitlinie Prostatakarzinom (Version 8) nicht mehr grundsätzlich zur Biopsie empfohlen, was von unseren Ergebnisse bestätigt wird [22]. Hier wird ein risikoadaptierter Ansatz für PI-RADS-3-Läsionen empfohlen, bei dem die PSA-Dichte als entscheidender Parameter für die Indikationsstellung einer Biopsie herangezogen wird. Dieser Algorithmus könnte künftig auch Einfluss auf die Auswahl des geeigneten Biopsieverfahrens haben. Die signifikant höhere Übereinstimmung zwischen Biopsiehistologie und endgültiger Resektathistologie bei PI-RADS-5-Läsionen im Vergleich zu PI-RADS-3-Läsionen (*p* = 0,029) belegt die steigende diagnostische Genauigkeit mit zunehmender Bildgebungssicherheit.

## Limitationen

Diese retrospektive, monozentrische Studie ist durch ihr Design und die Kohortengröße limitiert, was die Validität einschränkt. Ein direkter Vergleich von Biopsie- und Resektathistologie ist nur eingeschränkt möglich, da Unterschiede in Klassifikationssystemen, Interobservervariabilität bei der Gleason-Bewertung sowie die Beteiligung mehrerer diagnostischer Instanzen das Risiko systematischer Abweichungen erhöhen. Eine weitere Limitation der Studie besteht darin, dass der direkte Vergleich zwischen MRT-Fusionsbiopsie und systematischer Biopsie durch das retrospektive Studiendesign methodisch eingeschränkt ist. Durch die gleichzeitige Durchführung beider Verfahren kann die systematische Biopsie potenziell durch die MRT-Fusionsbiopsie beeinflusst werden, was zu einer teilweisen Abhängigkeit der Ergebnisse führen kann. Die geringere Zahl an Patienten mit verfügbarer RPE-Histologie limitiert die Aussagekraft. Prospektive, multizentrische Validierungen sind erforderlich, um die Generalisierbarkeit der Ergebnisse zu sichern.

## Schlussfolgerung

Das kombinierte Biopsieverfahren aus MRT-Fusions- und systematischer Biopsie detektiert csPCa signifikant häufiger als die jeweiligen Einzelmethoden und führt zu einer höheren Rate kurativer Therapieempfehlungen. Nach unseren Daten resultiert dieser Effekt jedoch nur in einem geringen absoluten Zugewinn von 3 % gegenüber der MRT-Fusionsbiopsie allein („number needed to test“ = 33), was den routinemäßigen zusätzlichen Einsatz der systematischen Biopsie im Rahmen der Primärdiagnostik als nicht zwangsläufig notwendig erscheinen lässt.

Die MRT-Fusionsbiopsie zeigte eine höhere Übereinstimmung mit der postoperativen Histologie als die systematische Biopsie, wobei der Unterschied statistisch nicht signifikant war. Hochgradige Tumoren der Gruppe Gleason ≥ 4 + 4 = 8 werden sowohl hinsichtlich Sensitivität als auch positivem prädiktivem Wert unzureichend erfasst. Insbesondere bei PI-RADS-3-Läsionen zeigt sich eine geringe diagnostische Sicherheit. Das diagnostische Potenzial könnte zukünftig durch den ergänzenden Einsatz hochauflösenden Mikroultraschalls weiter gesteigert werden [14].

## Fazit für die Praxis

- Die Kombination aus MRT-Fusions- und systematischer Biopsie erhöht den Anteil kurativer Therapieempfehlungen.
- Der absolute Zugewinn gegenüber der MRT-Fusionsbiopsie allein betrug 3 % („number needed to test“ = 33).
- Der routinemäßige Einsatz der systematischen Biopsie in der Primärdiagnostik sollte abgewogen werden.
- PI-RADS-3-Läsionen (Prostate Imaging Reporting and Data System) zeigen eine geringe diagnostische Treffsicherheit und sollten nicht routinemäßig biopsiert werden.

## Data Availability

Die Rohdaten und Dateien dieser Studie befinden sich auf einer verschlüsselten Festplatte am Universitätsklinikum Schleswig-Holstein (UKSH) und sind aufgrund des Schutzes patientenbezogener Daten nicht öffentlich zugänglich. Nach Rücksprache mit der Ethikkommission können die Daten auf begründete Anfrage bei den Autoren prinzipiell eingesehen werden. Es liegen keine Drittanbieterdaten oder zusätzlichen Materialien vor
